# Sonographic evaluation of retained products of conception within 48 h following delivery: a retrospective cohort study

**DOI:** 10.1007/s00404-024-07688-8

**Published:** 2024-08-12

**Authors:** Nadine Ashkar Majadla, Raneen Abu Shqara, Safaa Haj, Inshirah Sgayer, Nadir Ghanem, Lior Lowenstein, Marwan Odeh

**Affiliations:** 1 Raya Strauss Wing Department of Obstetrics and Gynecology, Galilee Medical Center, Nahariya, Israel; 2 Azrieli Faculty of Medicine, Bar Ilan University, Safed, Israel

**Keywords:** Retained placenta, Post-partum hemorrhage, Asherman’s syndrome, Retained products of conception

## Abstract

**Objective:**

Early diagnosis of retained products of conception (RPOC) is critical for directing clinical management and for preventing associated complications. This study aimed to evaluate the utility of post-delivery ultrasound in patients with risk factors for RPOC.

**Study design:**

A retrospective cohort-study was conducted in a single tertiary university-affiliated hospital (January 2016–September 2022). Sonographic evaluation, including endometrium thickness measurement and color Doppler, were reviewed of women with risk factors for RPOC: postpartum hemorrhage, a hemoglobin drop > 4 g/dl, manual removal of the placenta, and suspicious placenta. Results of early postpartum ultrasound (within 48 h), misoprostol administration and hysteroscopies were evaluated.

**Results:**

Of the 591 women included, RPOC was suspected in 141 (24%). Endometrial thickness > 5 mm was associated with sonographic RPOC diagnosis in 58%. Suspected sonographic RPOC was concluded for 100%, 92% and 7% of the women with marked, moderate, and undetectable vascularity, respectively, *p* < 0.001. Misoprostol 1000 mcg per rectum (PR) was administered to 86% of those with suspected RPOC; only 11% of them needed an operative hysteroscopy for removal of the RPOC. RPOC on a pathology report was confirmed for 71% of those who underwent hysteroscopy.

**Conclusions:**

Postpartum transabdominal ultrasonography within 48 h of delivery was effective for assessing RPOC. For appropriate triage, color Doppler grading increased the accuracy of RPOC diagnosis. Misoprostol treatment was successful in 88% of women with suspected sonographic RPOC. The combination of sonographic evaluation and misoprostol treatment for suspected RPOC might lower the rate of unnecessary invasive procedures.

## What does this study add to the clinical work

**Table:** 

Postpartum transabdominal ultrasonography within 48 hours of delivery was effective for assessing retained products of conception.	
In women with suspected retained products of conception, the combination of sonographic evaluation and misoprostol treatment might lower the rate of unnecessary invasive procedures.	

## Introduction

The term “retained products of conception” (RPOC) refers to intrauterine trophoblastic tissue that develops after conception, and persists after medical or surgical pregnancy termination, miscarriage, and vaginal or cesarean delivery [1]. RPOC complicates nearly 1% of all pregnancies [2]. The diagnosis of RPOC is essential yet challenging. Clinically, RPOC symptoms in the short term include vaginal bleeding, fever, and abdominal or pelvic pain. In the long term, RPOC may cause intrauterine adhesions, leading to an entity called “Asherman’s syndrome” [3]. Among common presentations of Asherman’s syndrome are hypomenorrhea and amenorrhea, infertility, and recurrent miscarriages [1, 4, 5]. RPOC is suspected following certain sonographic ultrasound and Doppler findings after termination of pregnancy, miscarriage or birth. The findings include an endometrial mass or increased endometrial thickness; and an echogenic, hypoechoic or mixed echo intracavitary pattern, together with high endometrial vascularity [6, 7]. Normal sonographic findings might obviate the need to explore the uterine cavity. However, a questionable sonographic finding might lead to unnecessary surgical intervention. In their prospective study, Ben-Ami et al. revealed a higher detection rate of RPOC, as confirmed by histologic examination, among women for whom combined clinical and Doppler sonography led to suspicion of retained trophoblastic tissue [2]. Doppler studies have been shown to be helpful in diagnosing RPOC. However, data on the value of Doppler in this context are sparse, and the absence of blood flow does not exclude the diagnosis of RPOC [8, 9]. Some have suggested hysteroscopy as the most reliable means of diagnosing RPOC [10].

Treatment of RPOC has evolved from blind curettage, which was once the standard treatment, to operative hysteroscopy. Curettage might damage the endometrial basal layer and consequently lead to intrauterine adhesions. Nowadays, curettage is less performed for this indication, while hysteroscopy is increasingly performed [10]. Compared with blind dilatation and curettage, operative hysteroscopy was reported to be associated with greater surgical success and with fewer incomplete evacuations and postoperative formations of intrauterine adhesions [11, 12]. The use of miniaturized instruments facilitates completing procedures that are subject to challenging circumstances such as the presence of cervical stenosis [13]. However, controversy remains as to whether reproductive outcomes improve following RPOC treatment by operative hysteroscopy compared to blind dilatation and curettage [14]. In a retrospective study, the time to conception was shorter after hysteroscopy than after dilatation and curettage and new infertility problems occurred less frequently [15]. On the other hand, according to a systematic review, reproductive outcomes were similar to those with dilatation and curettage [14].

The means of assessing RPOC and the defining criteria vary widely. A false diagnosis inevitably leads to curettage and possible complications. Cut‐off values of the endometrial thickness measurement that defines RPOC were reported in the range of 5–30 mm [16, 17]. A meta‐analysis on the diagnostic accuracy of endometrial thickness of 15 mm or more did not yield solid results [16]. A workable standard for establishing RPOC following delivery is imperative. In this retrospective study, we aimed to evaluate the diagnostic utility of post-delivery ultrasound, within 48 h of delivery, in women with risk factors for RPOC. We also evaluated the success rate of medical treatment with misoprostol and the need for hysteroscopy among women with postpartum suspected RPOC based on early transabdominal postpartum ultrasound.

## Materials and methods

### Study description

This retrospective cohort study was based on data that were recorded in a single tertiary university-affiliated hospital, Galilee Medical Center, Nahariya, Israel, from January 2016 to September 2022. Included were women in our department who underwent post-delivery ultrasound for suspected RPOC for the following indications: postpartum hemorrhage, a hemoglobin drop > 4 g/dl during the 48 h after delivery, manual removal of the placenta, and a suspicious placenta with a missing cotyledon, as suspected by a midwife or a physician. Excluded were women with RPOC after a missed abortion, an incomplete abortion or termination of pregnancy, or with uterine anomalies. The Institutional Review Board of Galilee Medical Center approved the study (Number 0150–22-NHR).

### Ultrasound studies

All sonographic evaluations were performed within 48 h of delivery by a specialist ultrasound physician in the maternal–fetal ultrasound unit in the Galilee Medical Hospital, using a transabdominal sonographic approach. In each examination, the endometrial thickness was measured, and a Doppler study was performed. The presence of a color Doppler signal and the amount of endometrial vascularity were assessed as undetected, minimal, moderate or marked. Undetectable vascularity was defined by an avascular color Doppler appearance. Minimal vascularity was defined as some detectable color Doppler flow in the endometrium but less than in the myometrium in the same image section. Moderate vascularity was defined as vascularity equal to or near equal to that in the myometrium in the same image section. Marked vascularity was defined as marked endometrial vascularity greater than that in the myometrium in the same image section [18]. Endometrial thickness was measured in a longitudinal view, and included the maximum anteroposterior diameter of the uterine cavity at the site of the suspected RPOC. Endometrial thickness was measured in millimeters and categorized as either ≥ 5 mm or < 5 mm. Finally, based on the ultrasound specialist’s overall impression of the sonographic findings, the examination was reported as “suspected sonographic RPOC” or “normal ultrasound”.

### Treatment of the RPOC protocol

Women with suspected RPOC of less than 30 mm, were offered a trial of misoprostol 1000 per rectum (PR). An exception was those with endometrial thickness of < 10 mm and with undetected or minimal vascularity on color Doppler, who were managed expectantly. Finally, those with endometrial thickness more than 30 mm underwent hysteroscopy as first-line treatment.). This was followed by sonographic evaluation after 2 weeks, for the presence of RPOC. In those with suspected RPOC not treated by misoprostol, and in those with a failed trial of misoprostol, a hysteroscopy was performed at 4–6 weeks after delivery. In light of the controversy regarding the optimal timing of post-delivery hysteroscopy for RPOC [19], this delay was intended to increase the possibility of a spontaneous resolution, and decrease the risks of uterine perforation, infection and larger spillage of distension medium. The latter may develop due to uterus enlargement and cervical dilation that presents soon after delivery, especially of full-term pregnancies [3].

### Study outcomes

We searched the database of our ultrasound unit for women who performed post-delivery ultrasound and fulfilled the study criteria. The charts were subsequently reviewed by the researchers; and demographics, ultrasound findings, intervention (misoprostol treatment and hysteroscopy performance) and complications were retrieved.

The primary study outcome was the diagnosis of RPOC in hysteroscopy. The secondary outcomes were postpartum results, including fever > 38 °C, endometritis, the need for blood transfusion or intravenous iron supplementation, the need for misoprostol administration, and the need for hysteroscopy. These outcomes, as well as demographic and clinical characteristics, were compared between women with suspected sonographic RPOC and women with normal ultrasound. We also analyzed correlations of color Doppler vascularity of the endometrium and of endometrial thickness > 5 mm, with the conclusion of “suspected sonographic RPOC” and with the need for hysteroscopy.

### Statistical analysis

Continuous variables are presented as means ± standard deviations, or as medians and ranges. Qualitative variables are presented as frequencies and percentages. Continuous variables were compared using either the independent sample *t*-test or the Mann–Whitney test, based on the sample sizes of the groups and the distribution shapes of the variables. Categorical variables were analyzed using Pearson’s chi-squared test or Fisher’s exact test. Statistical analysis was performed using IBM SPSS Statistics for Windows, version 27.0 (IBM Corp., Armonk, NY, USA).

## Results

During the study period, 591 women were examined in our maternal–fetal ultrasound unit due to suspected RPOC. Of them, 450 (76.1%) had normal sonographic findings and 141 had suspected postpartum RPOC. The proportion of women with a history of RPOC was significantly higher among those with a suspected sonographic RPOC (9%) than among those with a normal ultrasound (3%), *P* = 0.006. Maternal age, pregnancy number, parity, previous CS, delivery week, delivery by CS or vacuum, birthweight, and hemoglobin level before delivery were similar between the groups (Table 1).

**Table 1:** Characteristics of women according to sonographic results: suspected retained products of conception (RPOC) and normal gynecologic ultrasound

	Suspected sonographic RPOC (*n* = 141)	Normal ultrasound (*n* = 450)	*P* value
Maternal age, years	30.3 ± 5.7	29.4 ± 5.3	0.085
Pregnancy number	2 (1–11)	2 (0–12)	0.449
Parity	1 (0–8)	1 (0–8)	0.882
Previous CS	13 (9)	61 (14)	0.192
Number of previous CS	1 (0–8)	1 (0–8)	0.882
History of RPOC	12 (9)	13 (3)	0.006
Delivery week	40 (25.5–41.6)	39.5 (24–42)	0.010
Delivery by CS	9 (6)	52 (12)	0.082
Delivery by vacuum	1 (1)	12 (3)	0.320
Birthweight	3430 ± 509	3339 ± 491	0.059
Hemoglobin, g/dl before delivery	12.0 ± 1.50	11.9 ± 1.3	0.395

The data are presented as mean ± std, *n* (%), or median (range)

*CS* cesarean section

**Fig. 1:**
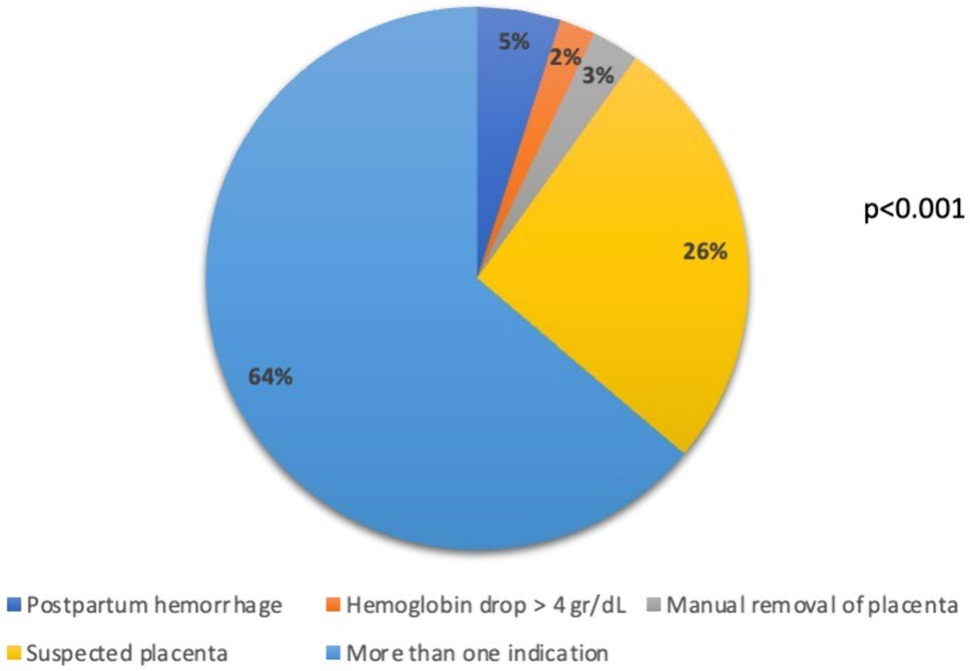
Indications for performing post-delivery ultrasound for suspected retained products of conception

The indications for ultrasound studies due to suspected RPOC were as follows: 36 (26%) due to suspicious post-delivery placenta, 7 (5%) due to postpartum hemorrhage, 4 (3%) due to manual removal of placenta and 3 (2%) due to a hemoglobin drop > 4 g/dl. More than one indication was observed in 90 (64%) women (Fig. 1).

### Correlations of Doppler findings with the detection of sonographic RPOC and the performance of hysteroscopy

Suspected sonographic RPOC was concluded for 7/7 (100%) women with marked vascularity, 37/ 40 (92%) with moderate vascularity, and 64/67 (95%) with minimal vascularity. However, only 33 (7%) with undetectable vascularity were diagnosed with suspected sonographic RPOC, *P* < 0.001 (Table 2). Hysteroscopy for RPOC was performed in 3 (43%) of those with marked vascularity on Doppler studies, 4 (1%) of those with moderate vascularity, 5 (7%) with minimal vascularity and 7 (1%) of those with undetectable vascularity, *P* < 0.001 (Table 2).

**Table 2:** Sonographic features related to the diagnosis of retained products of conception (RPOC) on ultrasound

Correlation of Doppler findings with the detection of sonographic RPOC on ultrasound			Correlation of Doppler findings with the need to perform hysteroscopy for RPOC		
	Suspected sonographic RPOC, *n* (%)	*P* value		Hysteroscopy performance *n* (%)	*P* value
Avascular(1)	33/477 (7)	< 0.001	Undetectable vascularity (1)	7/477 (2)	< 0.001
Minimal vascularity(2)	64/67 (96)		Minimal vascularity (2)	5/67 (7)	
Moderate vascularity(3)	37/40 (93)		Moderate vascularity (3)	4/40 (1)	
Marked vascularity(4)	7/7 (100)		Marked vascularity (4)	3/7 (43)	

Correlation of endometrium thickness ≥ 5 mm with the detection of sonographic residua on ultrasound			Correlation of endometrium thickness ≥ 5 mm with the need to perform hysteroscopy for RPOC		
	Suspected sonographic RPOC *n* (%)	*P* value		Hysteroscopy Performance *n* (%)	*P* value
Endometrium thickness ≥ 5 mm	81/191 (58)	< 0.001	Endometrium thickness ≥ 5 mm	12/191 (6)	0.003
Endometrium thickness < 5 mm	60/400 (15)		Endometrium thickness < 5 mm	7/400 (2)	

(1) Undetectable vascularity—in the endometrium

(2) Minimal vascularity—flow in the endometrium but less than in the myometrium

(3) Moderate vascularity equal to or near equal to that in the myometrium

(4) Marked vascularity—marked endometrial vascularity greater than that in the myometrium

### Correlations of endometrium thickness ≥ 5 mm and the detection of sonographic RPOC with the performance of hysteroscopy

Our data show that 60% of women with endometrium thickness ≥ 5 mm had a suspected sonographic RPOC (*P* < 0.001); otherwise, 60 of 400 (15%) women with an endometrium thickness < 5 mm had a suspected sonographic RPOC, *P* < 0.001 (Table 2). In a subgroup analysis, 6% of the women with an endometrium thickness ≥ 5 mm required a hysteroscopy for RPOC, compared to only 2% of those with an endometrium thickness < 5 mm, *P* < 0.001 (Table 2).

### Postpartum results of the study cohort

Postpartum clinical findings of women with suspected sonographic RPOC compared to women with normal ultrasound are presented in Table 3. The groups were similar in the mean hemoglobin level after delivery; and in the proportions with a hemoglobin drop > 4 g/dl, with fever > 38 °C, with endometritis, treated with intravenous iron, and recipients of a blood transfusion.

**Table 3:** Postpartum results of women with suspected residua on ultrasound vs. normal gynecologic ultrasound

	Suspected sonographic RPOC (*n* = 141)	Normal ultrasound (*n* = 450)	*P* value
Hemoglobin after delivery, mean ± std	8.6 ± 1.8	8.6 ± 1.9	0.927
Hemoglobin drop > 4 g/dl, mean ± std	3.4 ± 1.9	3.3 ± 1.8	0.474
Fever > 38 °C	16 (11)	29 (7)	0.067
Endometritis	3 (2)	9 (2)	1.000
Iron intravenous	73 (52)	196 (44)	0.081
Blood transfusion	14 (10)	41 (9)	0.868
Hospitalization length, median (range)	3 (0–15)	3 (0–30)	0.113

The data are presented as *n* (%) unless stated otherwise

*Std* standard deviation, *RPOC* retained products of conception

### Treatment by misoprostol and hysteroscopy performance

Of the 141 women with suspected sonographic RPOC, 121 (86%) underwent a trial of treatment by misoprostol 1000 mcg PR, 19 (14%) were managed expectantly, and one underwent a scheduled hysteroscopy. Of the 121 treated by misoprostol, 14 (12%) needed a hysteroscopy to remove suspected RPOC. Among the 14 with suspected sonographic RPOC who underwent hysteroscopy for suspected RPOC, pathology reports confirmed RPOC for 10 (71%). No complications were reported in women who underwent hysteroscopy.

## Discussion

Sixty-eight percent of the women diagnosed with sonographic RPOC in our department over an 8-year period had more than one clinical risk factor. Of those with marked vascularity on Doppler studies, 43% underwent a hysteroscopy for RPOC. Of women with suspected sonographic RPOC, 121 (86%) underwent a trial of treatment by misoprostol 1000 mcg PR, of whom 14 (12%) needed hysteroscopy for removal of suspected residua. Pathology reports confirmed RPOC for 71% of those who underwent hysteroscopy for suspected RPOC.

Among our patients with suspected RPOC, the most common indication that resulted in early postpartum RPOC sonographic diagnosis was a post-delivery suspicious placenta that was diagnosed by a midwife or an obstetrician. Suspected sonographic RPOC was determined for 26% of the women with this finding. RPOC was most commonly (64%) diagnosed following at least two indications. This suggests that the index of suspicion for RPOC should be raised in the presence of at least two of the following risk factors: postpartum hemorrhage, a postpartum hemoglobin drop > 4 gr/dl, a suspicious placenta and manual removal of the placenta.

A distinctive feature of our study is the exclusive inclusion of women with postpartum RPOC. Our findings corroborate a retrospective study of women with manual removal of placenta, in which routine elective sonography yielded a threefold higher rate of RPOC compared to evaluation of only symptomatic women [20].

We report a positive pathology report for 71% of our patients with suspected sonographic RPOC who underwent hysteroscopy. This is similar to the 67% rate reported previously [21]. The predictive value of sonographic RPOC following delivery is controversial. Smorgick et al. [22] concluded that the diagnosis of RPOC should be based on the presence of an echogenic mass on a sonographic scan, with positive Doppler flow. They reported 82% confirmation of RPOC during hysteroscopy among women considered at-risk. We report that in earlier postpartum ultrasound (< 48 h post-delivery), marked vascularity was correlated with a suspected sonographic RPOC and with the need for hysteroscopy after failed misoprostol treatment. This is in accordance with previous reports [18, 23]. On the other hand, a small proportion (6%) of women with endometrial thickness ≥ 5 mm required hysteroscopy for removal of RPOC. Although this rate was substantially higher (*P* = 0.003) than among those with endometrial thickness < 5 mm (2%), the rates were considerably lower than the 76% rate previously reported when the criteria for suspected RPOC was endometrial thickness > 7 mm [18, 23]. Other studies evaluating clinical parameters for diagnosis reported that the combination of endometrial hyperechogenic mass and clinical parameters such as pain and bleeding were not predictive of RPOC [17].

For a substantial proportion of women with suspected RPOC on ultrasound, the natural history is resolution with no need for treatment. Hence, an approach of watchful waiting is often warranted in the absence of symptoms. Previously, misoprostol for treatment of post-miscarriage RPOC was described as effective in about 76% [24]. However, we suggest that misoprostol should be considered as a second-line treatment for post-delivery suspected sonographic RPOC. This is in light of our finding that only 12% of those with suspected sonographic RPOC treated by misoprostol needed a hysteroscopy for the definitive treatment of RPOC.

A strength of our study is the relatively large sample. All sonography was performed by a specialist ultrasound physician. The proposal to use a postpartum sonography scan with the above-described sonography, combined with clinical parameters, was practical and easily implemented. We present a protocol for post-delivery RPOC evaluation and management that could be feasible in other regions.

Our study is limited by its retrospective design. Therefore, standardization of the examinations and of RPOC diagnosis was not possible. RPOC was suspected based on impressions of the ultrasound specialists; thus, operator bias could not be ruled out. Side effects of misoprostol and patient satisfaction were not reported. RPOC may resolve spontaneously; however, misoprostol treatment was administered to most of our patients diagnosed with suspected RPOC. This precluded assessing those who indeed needed this treatment. Finally, long-term follow-up of future fertility and the occurrence of Asherman’s syndrome were lacking.

The effect of misoprostol on RPOC following delivery should be examined in prospective studies. A possible research direction is triaging women according to clinical, sonographic and other potential biochemical markers. The value of various cut-offs for endometrial thickness should also be the focus of future studies.

## Conclusion

Postpartum transabdominal ultrasonography within 48 h of delivery was effective for assessing RPOC. For appropriate triage, color Doppler grading increased the accuracy of RPOC diagnosis. Misoprostol treatment was successful in 88% of the suspected sonographic RPOC. In women with suspected RPOC, the combination of sonographic evaluation and misoprostol treatment might lower the rate of unnecessary invasive procedures.

## Data Availability

The datasets generated during and/or analysed during the current study are not publicly available due to ethical restrictions but are available from the corresponding author on reasonable request.
